# Acute Hemiparesis in a Child as a Presenting Symptom of Hemispheric Cerebral Proliferative Angiopathy

**DOI:** 10.1155/2013/920859

**Published:** 2013-02-12

**Authors:** J. J. Gold, J. R. Crawford

**Affiliations:** ^1^Division of Child Neurology, Department of Neurosciences, University of California, San Diego and Rady Children's Hospital, 8010 Frost Street, Suite 400, San Diego, CA 92123, USA; ^2^Department of Pediatrics, University of California, San Diego and Rady Children's Hospital, 3020 Children's Way, San Diego, CA 92123, USA

## Abstract

A 9-year-old girl with a several-month history of unilateral intermittent headaches presented to the hospital with worsening headaches and unsteadiness. Neurologic exam was positive for a mild right hemiparesis and right homonymous hemianopsia. Noncontrast computed tomography revealed an engorged sagittal and straight sinus with prominent cortical veins concerning an arteriovenous malformation and the patient was admitted to the pediatric intensive care unit. Computed tomography angiogram demonstrated a left hemispheric vascular malformation, without evidence of dural arteriovenous fistula on conventional angiogram consistent with a diagnosis of cerebral proliferative angiopathy. There was no evidence of infarct on magnetic resonance imaging, and the patient's symptoms were completely resolved within 24 hours. Cerebral proliferative angiopathy is a rare but important vascular malformation distinct from classic arteriovenous malformations that may present with stroke-like symptoms in childhood.

## 1. Introduction

Cerebral proliferative angiopathy (CPA) has been demonstrated to be a distinct entity from classic arteriovenous malformations (AVMs) with regard to clinical presentation, neuroimaging features, and natural history [1]. Most commonly discovered in adolescent and middle-aged females in association with seizures, headaches, and stroke-like symptoms, there are no case reports of cerebral proliferative angiopathy in the pediatric population. We present a case of a young child with stroke-like symptoms in association with hemispheric CPA, not amenable to neurointerventional repair.

## 2. Case Presentation

**Figure 1:**
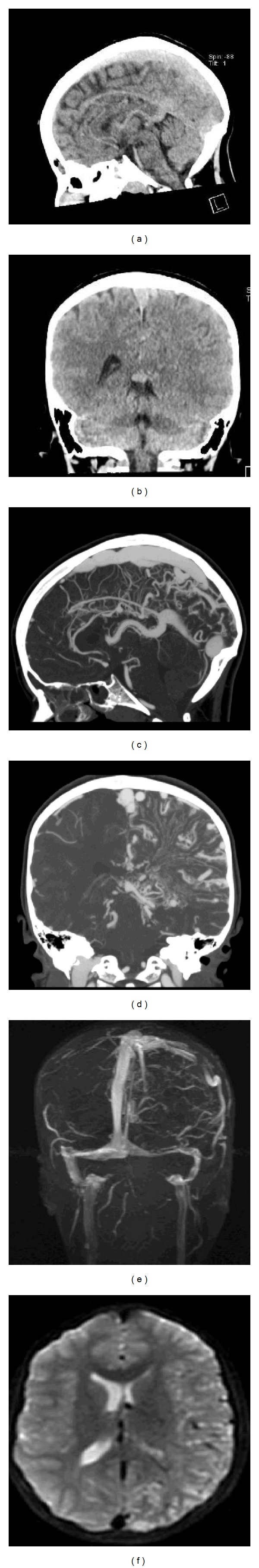
Noncontrast computed tomography in (a) sagittal and (b) coronal view revealed prominent left cortical veins concerning an AVM. CT angiography in (c) sagittal and (d) coronal view confirmed the diagnosis of left hemispheric cerebral proliferative angiopathy. Magnetic resonance venogram (e) showed the lesion and confirmed the absence of dural AV fistula. (f) Diffusion-weighted MRI showed no areas of ischemia.

A 9-year-old girl with a several-month history of unilateral headaches associated with nausea, vomiting, phonophobia, and photophobia presented to the emergency room for worsening headaches in association with acute clumsiness. General examination and vital signs were within normal limits. Neurologic exam was significant for a mild right hemiparesis and a complete right homonymous hemianopsia. Noncontrast computed tomography (CT) demonstrated an enlarged venous system with prominent left cortical veins concerning an AVM (Figures 1(a) and 1(b)), and the patient was admitted to the intensive care unit. CT angiography demonstrated a diffuse left hemispheric vascular malformation (Figures 1(c) and 1(d)) without evidence of a dural based AV fistula on conventional angiogram or magnetic resonance venography (Figure 1(e)) consistent with a diagnosis of CPA. Magnetic resonance imaging did not show evidence of acute ischemia on diffuse-weighted sequences (Figure 1(f)). The child did have an impressive left orbital bruit that was discovered in retrospect following the neuroimaging studies. Electroencephalogram showed diffuse high-voltage delta slowing throughout the left hemisphere without seizures. The child's motor and visual symptoms were completely resolved within 24 hours. However, she had multiple subsequent admissions for similar complaints over the next year, each without evidence of infarct or hemorrhage on MRI and each time with full resolution of neurologic symptoms within 24 hours in the absence of intervention.

## 3. Discussion

Differences in the underlying pathophysiology, natural history, clinical course, and recommended treatment distinguish cerebral proliferative angiopathy from “classic” AVM, making CPA an important clinical entity to identify [1]. One series reviewed a database of 1434 patients with AVMs and identified CPA in 49 (3.4%) of cases, with an age range of 10–65 years (median 17.5 years) [1]. The patient described in this case report was 9 years old at the time of symptom onset, which to our knowledge makes her the youngest patient described in the literature to date.

There have been relatively few case reports of CPA [2–7]. CPA is considered to have a low risk for hemorrhage at initial presentation, although a case of fatal hemorrhage has been described in an elderly woman [6]. Patients with CPA do not appear to have an increased incidence of other vascular abnormalities, although a case of patient with a hemangioma on the face and tongue ipsilateral to the brain lesion has been described [5]. As the name suggests, the malformation is proliferative, continuing to grow in all reported patients with neuroimaging at multiple time points [5, 7]. Treatment is challenging because the abnormal vasculature is interposed with normal cortex that is put at risk during repair, and early studies of the vascular lesion suggested that embolization might have disastrous consequences [3]. Nevertheless, embolization, radiotherapy, and vascular neurosurgery have all been attempted, with varying success [1, 4, 5, 7]. To our knowledge, this is the first detailed case report of a pediatric patient with CPA, which precludes us from drawing conclusions about the differences between pediatric and adult presentations of CPA.

CPA may be underrecognized in children. It is unclear when the malformation arises, but it has been shown that the lesion can progress over the span of several years [5, 7]. Early identification may be beneficial, because blood oxygen levels dependent on MRI imaging and MR perfusion imaging have suggested patients with CPA have low cerebrovascular reserve and are in a state of chronic hypoperfusion [3, 8]. CPA can be associated with a unilateral orbital bruit as was detected in our patient, which may improve detection in asymptomatic children. CPA is an important entity for neurologists to recognize it as a rare presenting feature of acute onset weakness in a child, whose symptoms may resolve in the absence of intervention.
